# Prognostic Role of Circulating Tumor Cells during Induction Chemotherapy Followed by Curative Surgery Combined with Postoperative Radiotherapy in Patients with Locally Advanced Oral and Oropharyngeal Squamous Cell Cancer

**DOI:** 10.1371/journal.pone.0132901

**Published:** 2015-07-17

**Authors:** Johanna Inhestern, Katrin Oertel, Viola Stemmann, Harald Schmalenberg, Andreas Dietz, Nicole Rotter, Johannes Veit, Martin Görner, Holger Sudhoff, Christian Junghanß, Claus Wittekindt, Katharina Pachmann, Orlando Guntinas-Lichius

**Affiliations:** 1 Department of Otorhinolaryngology, Jena University Hospital, Jena, Germany; 2 University Tumor Center, Jena University Hospital, Jena, Germany; 3 Department of ENT Surgery, University Medical Center Leipzig, Leipzig, Germany; 4 Department of Otorhinolaryngology, Head and Neck Surgery, Ulm University Medical Center, Ulm, Germany; 5 Department of Hematology and Oncology, Academic Teaching Hospital Bielefeld, Bielefeld, Germany; 6 Department of Otorhinolaryngology, Head and Neck Surgery, Academic Teaching Hospital Bielefeld, Bielefeld, Germany; 7 Division of Medicine, Department of Hematology, Oncology and Palliative Medicine, University of Rostock, Rostock, Germany; 8 Department of Otorhinolaryngology, Head and Neck Surgery, University Hospital Giessen and Marburg, Giessen, Germany; 9 Clinic of Internal Medicine II, Division of Hematology and Internal Oncology, Jena University Hospital, Erlanger Allee 101, Jena, Germany; Deutsches Krebsforschungszentrum, GERMANY

## Abstract

**Background:**

The prognostic role of circulating tumor cells (CTCs) after induction chemotherapy using docetaxel, cisplatin and fluorouracil (TPF) prior to surgery and adjuvant (chemo)radiation in locally advanced oral squamous cell cancer (OSCC) was evaluated.

**Methods:**

In this prospective study, peripheral blood samples from 40 patients of the phase II study TISOC-1 (NCT01108042) with OSCC before, during, and after treatment were taken. CTCs were quantified using laser scanning cytometry of anti– epithelial cell adhesion molecule–stained epithelial cells. Their detection was correlated with clinical risk factors, recurrence-free (RFS) and overall survival (OS).

**Results:**

Before starting the treatment CTCs were detected in 32 of 40 patients (80%). The median number at baseline was 3295 CTCs/ml. The median maximal number of CTCs during treatment was 5005 CTCs/ml. There was a significant increase of CTCs before postoperative radiotherapy compared to baseline before 1^st^ cycle of IC (p = 0.011), 2^nd^ cycle of IC (p = 0.001), 3^rd^ cycle of IC (p = 0.004), and before surgery (p = 0.002), but not compared to end of therapy (p = 0.118). CTCs at baseline >median was also associated to risk of recurrence (p = 0.014). Maximal CTCs during therapy >median was more frequently observed in tumors of the oral cavity (p=0.022) and related to higher risk of death during follow-up (p = 0.028). Patients with CTCs at baseline >median value had significant lower RFS than patients with CTCs at baseline <median value (p = 0.025). Patients with maximal CTCs values >median during the complete course of therapy had a significantly lower OS than patients with values <median (p = 0.049). Finally, the multivariate analysis revealed that OS was significantly lower in patients with maximal CTCs during treatment higher than the median value (HR=6.151; CI: 1.244-30.420).

**Conclusions:**

Baseline CTCs and maximal CTCs during therapy both seem to be good prognostic markers for OSCC when treated by TPF induction chemotherapy, surgery, and postoperative (chemo)radiation.

## Introduction

About two thirds of patients with squamous cell carcinomas of the head and neck (SCCHN) present with advanced disease (Stage III/IV). About 50% of SCCHN sustain local recurrence and up to 25% develop distant metastases [1, 2]. All of this results in a relatively poor prognosis with a 5-year survival rate of approximately 50%. One of the important risk factors is detected or occult lymph node metastasis in the neck. In order for SCCHN to spread other than by direct extension from its primary site in the head and neck to regional lymph nodes, the tumor must gain access to lymphovascular channels [3]. During this process tumor cells seem to detach from their primary tumor and enter the peripheral circulation. These cells are designated circulating tumor cells (CTCs) and are able to deposit within lymph nodes and other organs where they may proliferate and develop into eventual metastatic tumors. A CTCs is an epithelial derived, nucleated, often cytokeratin-positive cell that is not a normal constituent of blood, and cannot be identified in patients without an epithelial malignancy [4]. The clinical relevance of CTCs detection in carcinoma patients has been part of extensive research, and encouraging results exist for an association between CTCs detection and the prognosis in patients with other solid tumors, such as metastatic breast, prostate, and gastrointestinal cancers [5–7]. In SCCHN patients CTCs might potentially serve as a marker for nodal/metastatic disease, for assessing treatment susceptibility, for post-treatment cancer surveillance, and for prognosis [3].

Induction chemotherapy (IC) is one established strategy to reduce the risk of recurrence. IC has regained interest in SCCHN by two hallmark phase III trials using docetaxel, cisplatinum and 5-fluorouracil (TPF) as IC in locally advanced SCCHN prior to radiotherapy [8]. Recently we could show in a phase I trial that TPF is also feasible prior to surgery of locally advanced oral squamous cell cancer (OSCC)[9]. Meanwhile, this setting was also proven in a phase III trial [10]. OSCC represent about more than half of all SCCHN [11]. The value of CTCs in an IC setting to treat OSCC remains undetermined. The primary aim of this prospective study was therefore to establish the prognostic role of CTCs in advanced non-metastatic OSCC treated by IC before surgery and postoperative radiotherapy.

## Materials and Methods

This translational study was part of an ongoing prospective multicenter phase II clinical trial (TISOC-1) conducted at six institutions in Germany (ClinicalTrials.gov; NCT01108042; 72 patients included; last patient in: August 2013; last patient out: December 2015). The first 50 patients took place in this translational study. The per-protocol follow-up of the 50^th^ patient ended in January 2014. Therefore, it was possible to report the outcome of this translational study before the phase II clinical trial is ended. All institutions included patients in the translational study on the prognostic role of CTCs. The translational study was coordinated in the Department of Otorhinolaryngology, University Hospital Jena, Germany. The protocol was centrally approved by the ethics committee for human research at the Medical Faculty, Friedrich-Schiller-University Jena, and adopted by the ethics committee of all study centers. All patients provided written informed consent before registration. Patients were included from February 2010 to May 2012 and have been observed until January 2014 or death.

### Patient eligibility

Patients aged ≥18 and ≤80 years were eligible for inclusion if a histologically confirmed resectable squamous cell carcinoma of the oral cavity (excluding the lip), or of the oropharynx had been diagnosed. Tumors were judged resectable when an R0 resection was likely to be achieved. Tumors were included if they were classified as any cTcN2M0, any cTcN3M0, cT3 and cN0-1M0, cT4 and cN0-1M0 (International Union Against Cancer). Furthermore, inclusion required a Karnofsky Index ≥70% without high anesthetic risk. Adequate pulmonary, cardiac, bone marrow, hepatic and renal functions were mandatory. Exclusion criteria were distant metastatic disease; a life expectancy of <3 months; pregnancy; previous cancer disease within 5 years of study entry. Furthermore, patients were not entered in case of serious concomitant diseases or serious medical conditions; previous treatment with chemotherapy, radiotherapy or surgery for head and neck cancer; or any social situations that would have limited the compliance with study requirements. Concurrent treatments with other experimental drugs or participation in another clinical trial with any investigational drug within 30 days before study screening were also not allowed.

### Study treatment

TISOC-1 started with a split-dose IC containing a combination of docetaxel (Taxotere, Sanofi-Aventis), cisplatin and 5-fluorouracil (split TPF). The safety and feasibility of the split TPF regime was proven in preceding phase I trial [9]. According to the results of the phase I study, the phase II study was conducted with a dose of 30 mg/m^2^ docetaxel plus 40 mg/m^2^ cisplatin and 2000 mg/m^2^ fluorouracil per week. Each chemotherapy cycle lasted 3 weeks. Chemotherapy was applied with usual premedication, appropriate antiemetics and intravenous hydration. Chemotherapy was given on days 1, 8 (cycle 1), and for responders (see below) further on day 22, 29 (cycle 2), and 43, 50 (cycle 3). Tumor response was evaluated on day 21 using a clinical examination with endoscopy of the primary tumor. Additionally, a CT or MRI of the neck with tumor volumetry was performed. Tumor response to chemotherapy was defined by the RECIST criteria and as a reduction of the tumor volume ≥ 30% [12]. Complete remission (CR) was defined as complete disappearance of primary tumor and neck metastasis. Partial response (PR) was defined as mentioned above but without complete disappearance. Progressive disease (PD) was defined as enlargement of tumor volume ≥ 20% or new tumor manifestations. Stable disease (SD) was fulfilled for tumors with enlargement <20% to reduction < 30%. Responders were patients with CR or PR. All responders were assigned to treatment arm B and received two more cycles of split-TPF, i.e. responders underwent surgery after three cycles of IC. Non-responders were assigned to treatment arm A and underwent surgery after one cycle of IC. Treatment cycles were repeated if the absolute neutrophil count (ANC) was ≥ 1.000/mm3, platelet count was ≥ 80.000/mm3, neurotoxicity ≤ grade 1, hand-foot-syndrome ≤ grade 1, and adequate kidney and liver function. If these criteria had not been recovered after a 2-week delay, chemotherapy was continued with a 70% reduction after recovery of the blood parameters. Surgery was performed within the original tumor margins prior to ICT. Standard histopathology was performed. p16 IHC staining was the surrogate marker for human papillomavirus (HPV). All p16 positive tumors were also tested for HPV DNA by PCR as described previously [13]. More details on the study protocol, postoperative treatment and evaluations were published recently [9].

### Detection of CTCs

CTCs were detected as previously reported [14]. Briefly, 7.5ml of blood anticoagulated with EDTA were drawn before starting the treatment with the first cycle of IC (baseline), before second and third cycle of IC, before surgery, before postoperative radio(chemo)therapy and at the end of treatment. Concerning the prognostic role of CTCs, the absolute amount of CTCs at baseline, every increase or decrease between each step of the treatment, and the highest value of CTCs of all measurements during the treatment (maximal CTC values) were analyzed. 1ml of each blood sample was lysed with ammonium chloride using 10 ml of erythrocyte lysis solution (Qiagen, Hilden, Germany) for 10 min at room temperature spun down at 700×g and re-diluted in 1 ml of PBS (phosphate-buffered saline) pH 7.4. For detection of CTCs, white cells from the sediment were subject to the MAINTRAC analysis, diluting the pellet in 500 μl of phosphate-buffered saline (pH 7.4) and adding 12.5 μl of fluorescein isothiocyanate–conjugated mouse anti-human epithelial antibody (Miltenyi Biotec, Bergisch Gladbach, Germany) and 4 μl of 7AAD (Sigma Aldrich, St.Louis, MO, USA), (50μg/ml) simultaneously for 15 minutes in the dark. Analysis of red and green fluorescence of the cells was performed using an Olympus Scan^R screening station which allows automated image acquisition and data analysis enabling relocation of cells for visual examination of vital epithelial cells as extensively described in a previous study [15]. A defined volume of the cell suspension was applied to each well of a microtiter plate and scanning was performed on the well area. Cells were detected in transmitted light, and red and green fluorescence was recorded. The epithelial cells selected by their green fluorescence were relocated and analyzed for vitality. Only vital cells were counted. CTCs were calculated per ml. It should be emphasized that the expression of anti-Human Epithelial Specific Antigen (ESA or epithelial cell adhesion molecule [EpCAM]) on the cell surface is not a proof that these cells are tumor cells. Although we have previously shown that EpCAM is not expressed on the surface of hematopoietic cells [16], this does not preclude that normal epithelial cells which have been disseminated into blood are detected by this approach.

### Statistical Analysis

Statistical analyses were performed using IBM SPSS version 21.0 statistical software for Windows (Chicago, Illinois, USA). To compare tumor characteristics or other parameters to the amount of detected CTCs at several time points of treatment or to compare other parameters, Pearson’s chi-square test was used. If necessary, the numeric CTC parameters were dichotomized to categorical data using median values as separators: To analyze the role of the number of CTCs at baseline, the study sample was divided into two groups (smaller the median value versus greater the median value of CTC at baseline). To analyze the role of the maximal number of CTCs during the course of therapy, the study sample was also divided into two groups (smaller the median value versus greater the median of the maximal number of CTC during the whole course of treatment). Unifactorial analysis of variance (ANOVA) with post-hoc Bonferroni correction was used to compare CTCs amounts at the different time points. Recurrence-free survival (RFS) and overall survival (OS) were calculated by the Kaplan-Meier method and differences of survival were compared by the log-rank test. RFS was defined as the elapsed time between the date of the first CTC examination at baseline prior to treatment and the date of tumor recurrence, or the last follow-up (if no death was observed during the follow-up period). OS was defined as the elapsed time between the date of the first CTC examination at baseline prior to treatment and either the date of death or the last follow-up (if death was not observed during the follow-up period). Multivariable analysis was performed using the Cox proportional hazards model including significant parameters from the log-rank tests to estimate the hazard ratio (HR) with a confidence interval (CI) of 95% for RFS and OS. For all statistical tests, significance was two-sided and set to p < 0.05.

## Results

### Patient’s, tumor and treatment characteristics

Clinicopathologic characteristics and treatment characteristics are summarized in Table 1 and Table 2. Most patients were male (83%). Median age was 58 years. The primary tumor was localized in the oral cavity in 15 patients (38%) and in the oropharynx in 25 patients (63%). The tumors of nineteen patients were HPV positive. Thirty-eight patients (95%) were clinically classified before treatment according to UICC stage IV and two patients (5%) as UICC stage III. Due to the tumor response after the first cycle of IC, 15 patients (38%) were treated in treatment arm A and 35 patients (63%) in treatment arm B. Surgery of the primary tumor was performed in all patients. All but one patient received a bilateral neck dissection, the one patient unilateral neck dissection. Histopathology revealed a complete remission of the primary tumor (ypT0) in 15 patients (38%) and complete remission of the neck metastases (ypN0) in 14 patients (35%). This led to a complete pathologic response in 8 patients (20%) and to a partial pathologic response in 24 patients (60%). All but one patient (96% of the arm) in treatment arm B revealed a complete or partial pathologic response, whereas in treatment arm A no patients showed a complete pathologic response and only 8 patients (53% of the arm) showed a partial pathologic response, i.e. the pathologic response to IC was significantly better in treatment arm B (p = 0.003). The most frequent postoperative treatment was radiotherapy (63%), followed by radiochemotherapy (30%), and radioimmunotherapy with cetuximab (18%).

**Table 1:** Clinicopathologic characteristics.

Parameter	Frequency (N)	%
Total number of cases	40	100
Gender		
Male	33	83
Female	7	18
Tumor site		
Oral cavity	15	38
Oropharynx	25	63
cT stage		
T2	15	38
T3	15	38
T4a	10	25
cN stage		
N0	3	8
N1	0	0
N2	35	87
N3	2	5
cM stage		
M0	42	100
M1	0	0
yT stage		
ypT0	15	38
ypT1	10	25
ypT2	4	10
ypT3	6	15
ypT4	5	13
ypN stage		
ypN0	14	35
ypN1	6	15
ypN2	20	60
	Mean±SD	Median, range
Age	58±8	58, 44–73

**Table 2:** Treatment characteristics.

Parameter	Frequency (N)	%
Total number of cases	40	100
Treatment arm		
A	15	38
B	25	63
Cycles of induction chemotherapy		
1	15	38
2	1	3
3	24	60
Pathologic response to induction chemotherapy		
Complete response (CR)	8	20
Partial response (PR)	24	60
Stable disease (SD)	6	15
Progressive disease (PD)	2	5
Surgery*		
Yes	40	100
No	0	0
Postoperative radio(chemo)therapy		
Radiotherapy	25	63
Radiochemotherapy	12	30
Radioimmunotherapy	3	18

*all surgeries were performed R0.

### Circulating tumor cells (CTCs)

**Fig 1:**
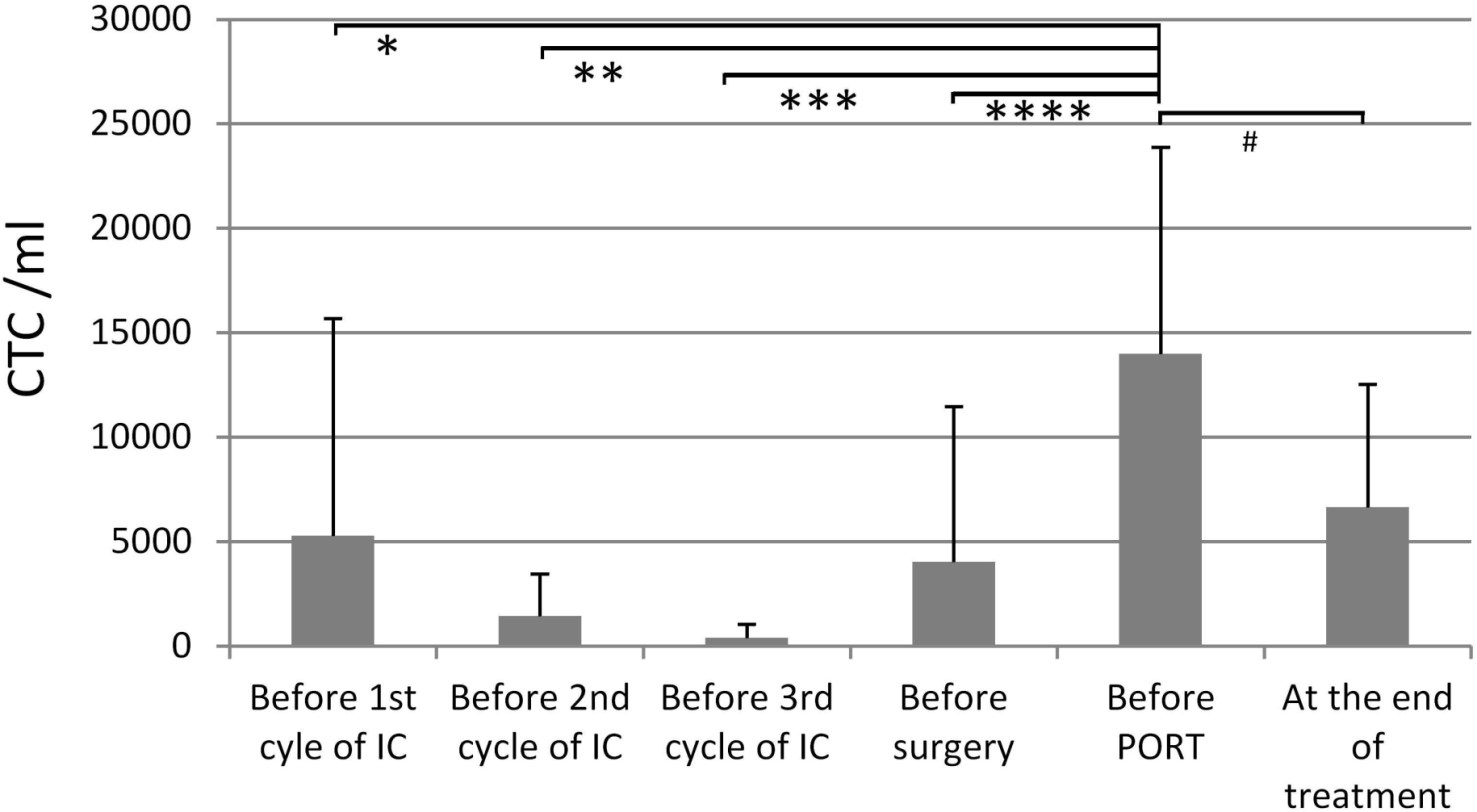
Number of circulating tumor cells per ml blood (CTCs/ml; mean ± standard deviation) before start of the treatment, during induction chemotherapy (IC), before surgery, before postoperative radiotherapy (PORT; ± chemotherapy or cetuximab), and at the end of treatment. Significant increase of CTCs before postoperative radiotherapy compared to baseline before 1^st^ cycle of IC (*; p = 0.011), 2^nd^ cycle of IC (**; p = 0.001), 3^rd^ cycle of IC (***; p = 0.004), and before surgery (****; p = 0.002), but not compared to the end of therapy (^#^; p = 0.118).

Only eight patients (20%) did not have any CTCs at baseline prior to treatment. The median number of CTCs at baseline was 3295/ml. On average, CTCs decreased during IC, but increased after surgery and finally reached values comparable to baseline (Fig 1). There was a significant increase of CTCs before postoperative radiotherapy compared to baseline before 1^st^ cycle of IC (p = 0.011), 2^nd^ cycle of IC (p = 0.001), 3^rd^ cycle of IC (p = 0.004), and before surgery (p = 0.002), but not compared to the end of therapy (p = 0.118). Median maximal CTC number during treatment was 5005 CTCs/ml. After treatment only one patient (3%) did not have any CTCs. More data on CTCs during the study treatment are presented in Table 3. Higher (>median) CTCs at baseline was associated to higher (>median) maximal CTCs during therapy (p = 0.023; S1 Table). Higher (>median) CTCs at baseline was also associated to recurrence (p = 0.014). Higher (>median) maximal CTCs during therapy was more frequently observed in tumors of the oral cavity (p = 0.022) and related to higher risk of death during follow-up (p = 0.028).

**Table 3:** Circulating tumor cells (CTCs) during TISOC1 treatment.

Parameter	Frequency (N)	%
Total number of cases	40	100
No CTCs at baseline	8	20%
No CTCs at the end of therapy	1	3%
	Mean±SD	Median, range
CTCs at baseline (CTCs/ml)	5266±10419	3295, 0–55650
CTCs before second cycle of neoadjuvant chemotherapy (CTCs/ml)	1241±2032	610, 0–6350
CTCs before third cycle of neoadjuvant chemotherapy (CTCs/ml)	382±644	0, 0–1710
CTCs before surgery (CTCs/ml)	4370±8229	853, 0–38670
CTCs before postoperative radiotherapy(CTCs/ml)	13991±9875	11600, 490–29620
CTCs, at the end of therapy (CTCs/ml)	6654± 5864	5370, 0–19640
CTCs, maximal (CTCs/ml)	9286±11298	5005, 0–55650

### Recurrence-free survival (RFS) and overall survival (OS)

The median follow-up time of all patients was 24.7 months (mean: 23.1±9.6 months). The median follow-up time of all patients alive at the end of the observation period was 29.8 months (mean: 26.1±8.6 months). No recurrence was observed in 32 patients (80%). Eight patients (20%) developed a recurrence. Thirty patients (75%) were alive and ten patients (25%) were dead at the end of the observation period.

**Fig 2:**
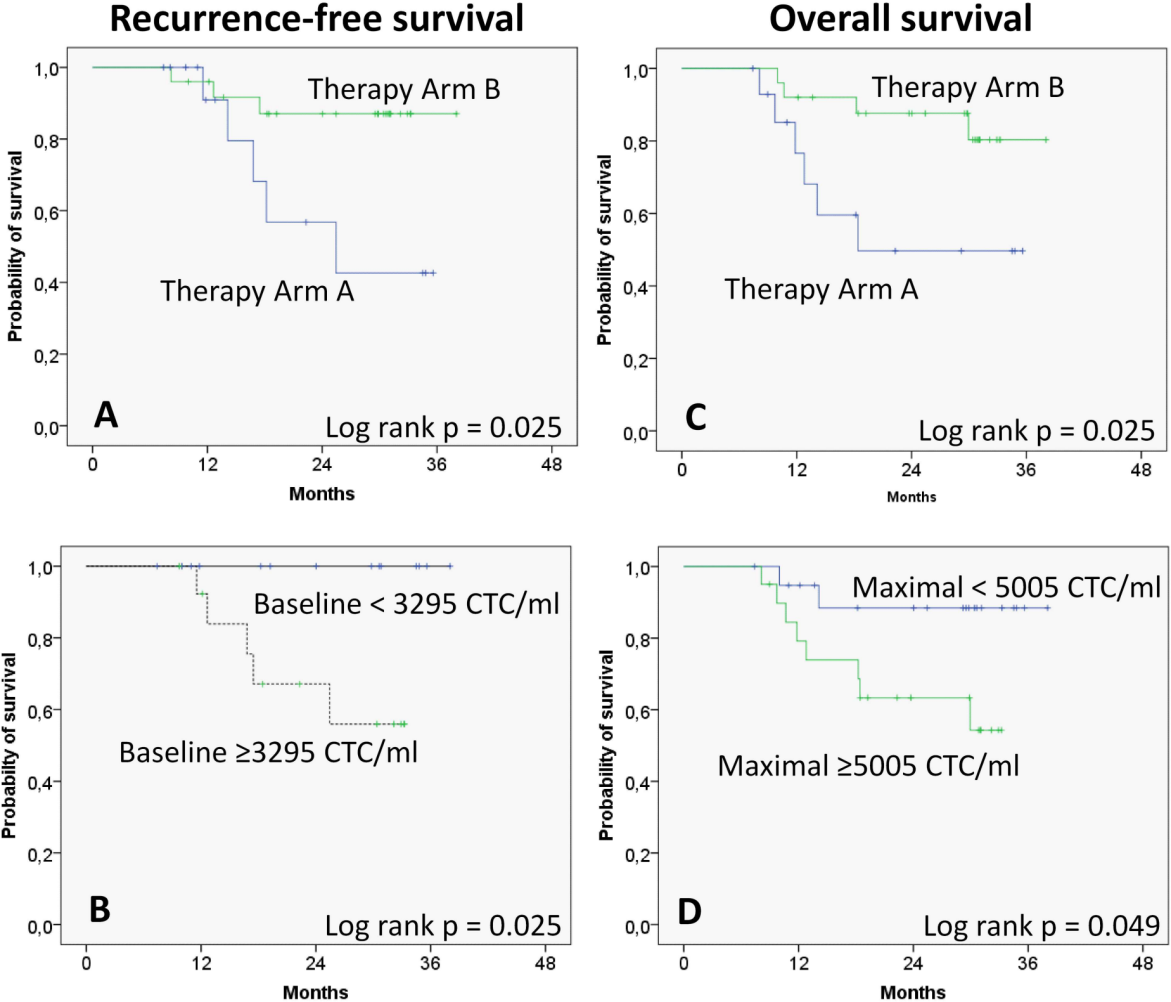
Kaplan-Meier curves on recurrence-free survival (A, B) and overall survival (C, D) according to therapy arm (A, C), circulating tumor cells (CTCs) at baseline (B), and maximal amount of CTCs during the treatment.

Overall, mean RFS was 32.4 months (95%CI: 29.0–35.8). 2-year RFS rate and 3-year RFS rate were 78.4% and 74.3%, respectively. Mean OS was 31.3 months (95%CI: 27.8–35.0). 2-year OS rate and 3-year OS rate were 75.1% and 70.1%, respectively. The results of the log-rank test are shown in Table 4. Patients in treatment arm A had a significantly lower RFS and OS than patients in arm B (p = 0.025 and p = 0.025, respectively; Fig 2A and 2C). Patients with CTCs at baseline >median value had significantly lower RFS than patients with CTCs at baseline <median value (p = 0.025; Fig 2B). Furthermore, patients with maximal CTCs values >median during the complete course of therapy had a significantly lower OS than patients with values <median (p = 0.049; Fig 2D). No other factors had a significant influence on RFS or OS in the univariate analysis. Finally, the multivariate analysis revealed that the treatment arm and maximal CTCs during the time course of therapy both were independent prognostic risk factors for OS (Table 5). OS was significantly lower for treatment arm A (Hazard ration [HR] = 5.758; CI: 1.503–22.059). For patients with maximal CTCs during treatment higher than the median value, OS was also significantly lower (HR = 6.151; CI: 1.244–30.420).

**Table 4:** Univariate analysis (log-rank test) of negative prognostic parameters influencing recurrence-free survival (RFS) and overall survival (OS).

Parameter	RFS	OS
Gender, female	0.219	0.951
Age (≥median of 58 years)	0.890	0.877
Localization, oral cavity	0.101	0.100
cT Stage (advanced, T3/T4)	0.409	0.591
cN Stage (N+)	0.675	0.963
HPV status (HPV negative)	0.270	0.791
Pathological response (not CR)	0.326	0.066
Therapy arm (Arm A)	**0.025**	**0.025**
CTCs at baseline, >median	**0.025**	0.951
CTCs maximal during therapy, >median	0.355	**0.049**
CTCs increase after IC	0.660	0.437
CTCs increase after surgery	0.367	0.188
CTCs increase after radiotherapy	0.385	0.787

CR = complete response; IC = induction chemotherapy

**Table 5:** Multivariable Cox regression of negative risk factors for recurrence-free survival (RFS) and overall survival (OS).

Factor		HR*	95%CI	p
**RFS**				
Therapy arm	B	1	Reference value	0.101
	A	4.541	0.744–27.716	
CTCs at baseline	<median	1	Reference value	0.950
	>median	0	0–1.509E+162	
**OS**				
Therapy arm	B	1	Reference value	**0.011**
	A	5.758	1.503–22.059	
CTCs maximal	<median	1	Reference value	**0.026**
	>median	6.151	1.244–30.420	

*HR = Hazard Ratio

## Discussion

Compared to other cancer entities, the impact of CTCs in SCCHN patients has not yet been extensively documented and is not applied in clinical routine [17]. It is known that the stage of disease is correlated to the CTC levels. In untreated local advanced SCCHN 12.5–100% of patients CTCs are detected depending on the detection method and included tumor stages [18–21].

The number of detected CTCs depends on the detection method [17, 20]. The FDA-approved automated CellSearch System (Veridex, New Jersey, USA) uses magnetically labeled EpCAM-specific antibodies to separate the adhering CTCs in a magnetic field. Using this approach, median CTCs numbers of 0–2 CTCs/7.5ml in locally advanced SCCHN are reported [22, 23]. Using a flow cytometric assay for the detection of CTCs, the number of CTCs ranged between 1-4/3.75 ml in locally advanced SCCHN [18, 21]. Using a multiparameter analysis based on a negative depletion enrichment methodology, baseline values of 0–3969 CTCs/ml are reported for locally advanced SCCHN [3].Using a negative selection method for effective leukocyte depletion and enhanced detection of viable EpCAM positive and negative CTCs, the number are in the same scale with 0–3440 CTCs/ml [24]. The method used in the present study is characterized by its non-dissipative approach and gives CTCs numbers in the same scale. The scanning microscope reliably detects the EpCAM positive events among the other blood cells without any additional enrichment step and allows visual inspection of the vital cells excluding the viability marker 7AAD. Repeated preparations of the same blood samples gave less than 10% variation in CTCs numbers and thus highly reproducible results. Using conventional blood count tubes and shipping at ambient temperature allowed easy sampling in a multicenter setting. Under these conditions samples showed >95% cell viability. Using EpCAM as marker does not preclude that normal epithelial cells which have been disseminated into blood are detected by this approach. However, since circulating epithelial cells were detected in 80% of patients with non-metastatic SCCHN and their number correlated with disease outcome the cells detected could be subsumed as CTCs.

It is important to note that OSCC does not represent a homogenous entity of HNSCC. In contrast to tumors of the oral cavity, oropharyngeal tumors, especially tonsil cancer, often is associated to human papillomaviruses (HPV) infection. This has influence on the carcinogenesis and normally also on the prognosis of the patients [25]. Interestingly, tumor localization and HPV status did not have a significant impact on OS under the complex treatment concept of present study. In the present study, response to IC was the most significant prognosticator for OS as it was shown by others when using IC in head and neck organ preservation protocols [26, 27]. A high number of CTCs during the course of the treatment was the other independent negative prognosticator for OS. Hence, the present study showed for the first time the impact of detection of changes of the number of CTCs during OSCC treatment which included IC. So far there was only some evidence that detection of CTCs at baseline correlates with worse RFS and OS in locally advanced HNSCC using surgery and postoperative radiotherapy or concurrent radiochemotherapy [21, 23, 28].

Looking on the time course of the CTCs detection during therapy, CTCs decreased on average during IC, re-increased before surgery, re-increased to maximal values after surgery and re-decrease after postoperative radiotherapy. So far not much is known about the time course of CTC detection during HNSCC therapy and its role as marker for treatment response. Most studies include heterogeneous groups of HNSCC or have a small sample size [17]. It has been shown that CTCs decrease after chemoradiation [22]. The data on the effect of surgery on CTCs is contradictory: CTCs seem to be significantly present after surgery [29], may even increase after surgical manipulation of head and neck tumors [30], but a decrease of CTCs after tumor resection has also be reported [18]. An increase of CTCs after tumor resection has also be reported for instance for esophageal cancer, lung cancer, or rectal cancer [31–33]. It must kept in mind for the present study, that surgery was performed after IC and that 20% of patients had a complete pathologic response, i.e. no more vital tumor cells were observed by standard histopathological methods in primary tumor area and the neck. Despite of this, CTCs increased in most of these patients after surgery. Paradoxically, the relative increase of CTCs after surgery had no negative prognostic impact on RFS or OS. In a recent study by Tinhofer et al. [29], the detection of CTCs after surgery and before adjuvant chemoradiation in patients with oropharyngeal cancer (in contrast to other HNSCC sites) even was associated per trend with improved disease-free survival. The reason is unclear and need to be elucidated in further studies with larger study samples.

It should not be neglected that IC is not considered to be part of standard therapy for locally advanced OSCC. Hence, patients should be carefully selected when considering IC. Recently, first predictive biomarkers for the response to IC like the TP53 mutation status, Cyclin D1 (CCND1) expression, or Annexin A1 expression have been reported [34–36]. If CTCs may also represent a potential predictive marker has to be explored in more detail in future studies.

In conclusion, we established the detection of CTCs as an independent prognostic markers for OSCC when treated by TPF induction chemotherapy, surgery, and postoperative (chemo)radiation. The method used allows a reliable collection of CTCs at different time points during therapy even in multicenter trial settings. Therefore, it seems to be feasible to validate our findings, and especially to examine more profoundly the role of CTCs as a marker for therapy response during IC, in a future larger trial.

## Supporting Information

S1 Table Univariate analysis of the association between CTC at baseline and maximal CTC during therapy, respectively, to other clinicopathologic characteristics. (DOCX)

## References

[1] Forastiere A, Koch W, Trotti A, Sidransky D (2001) Head and neck cancer. N Engl J Med 345: 1890–1900. 11756581 10.1056/NEJMra001375

[2] Argiris A, Karamouzis MV, Raben D, Ferris RL (2008) Head and neck cancer. Lancet 371: 1695–1709. 10.1016/S0140-6736(08)60728-X 18486742 PMC7720415

[3] Balasubramanian P, Lang JC, Jatana KR, Miller B, Ozer E, Old M, et al (2012) Multiparameter analysis, including EMT markers, on negatively enriched blood samples from patients with squamous cell carcinoma of the head and neck. PLoS One 7: e42048 10.1371/journal.pone.0042048 22844540 PMC3406036

[4] Schlimok G, Funke I, Holzmann B, Göttlinger G, Schmidt G, Häuser H, et al (1987) Micrometastatic cancer cells in bone marrow: in vitro detection with anti-cytokeratin and in vivo labeling with anti-17-1A monoclonal antibodies. Proc Natl Acad Sci U S A 84: 8672–8676. 2446326 10.1073/pnas.84.23.8672 PMC299608

[5] Cristofanilli M, Budd GT, Ellis MJ, Stopeck A, Matera J, Miller MC, et al (2004) Circulating tumor cells, disease progression, and survival in metastatic breast cancer. N Engl J Med 351: 781–791. 15317891 10.1056/NEJMoa040766

[6] Cohen SJ, Punt CJ, Iannotti N, Saidman BH, Sabbath KD, Gabrail NY, et al (2008) Relationship of circulating tumor cells to tumor response, progression-free survival, and overall survival in patients with metastatic colorectal cancer. J Clin Oncol 26: 3213–3221. 10.1200/JCO.2007.15.8923 18591556

[7] Scher HI, Jia X, de Bono JS, Fleisher M, Pienta KJ, Raghavan D, et al (2009) Circulating tumour cells as prognostic markers in progressive, castration-resistant prostate cancer: a reanalysis of IMMC38 trial data. Lancet Oncol 10: 233–239. 10.1016/S1470-2045(08)70340-1 19213602 PMC2774131

[8] Posner M, Vermorken JB (2008) Induction therapy in the modern era of combined-modality therapy for locally advanced head and neck cancer. Semin Oncol 35: 221–228. 10.1053/j.seminoncol.2008.03.007 18544437

[9] Oertel K, Spiegel K, Schmalenberg H, Dietz A, Maschmeyer G, Kuhnt T, et al (2012) Phase I trial of split-dose induction docetaxel, cisplatin, and 5-fluorouracil (TPF) chemotherapy followed by curative surgery combined with postoperative radiotherapy in patients with locally advanced oral and oropharyngeal squamous cell cancer (TISOC-1). BMC Cancer 12: 483 10.1186/1471-2407-12-483 23083061 PMC3485626

[10] Zhong LP, Zhang CP, Ren GX, Guo W, William WN Jr, Sun J, et al (2013) Randomized phase III trial of induction chemotherapy with docetaxel, cisplatin, and fluorouracil followed by surgery versus up-front surgery in locally advanced resectable oral squamous cell carcinoma. J Clin Oncol 31: 744–751. 10.1200/JCO.2012.43.8820 23129742 PMC5569675

[11] Guntinas-Lichius O, Wendt TG, Kornetzky N, Buentzel J, Esser D, Böger D, et al (2014) Trends in epidemiology and treatment and outcome for head and neck cancer: A population-based long-term analysis from 1996 to 2011 of the Thuringian cancer registry. Oral Oncol 50: 1157–1164. 10.1016/j.oraloncology.2014.09.015 25459063

[12] Eisenhauer EA, Therasse P, Bogaerts J, Schwartz LH, Sargent D, Ford R, et al (2009) New response evaluation criteria in solid tumours: revised RECIST guideline (version 1.1). Eur J Cancer 45: 228–247. 10.1016/j.ejca.2008.10.026 19097774

[13] Glombitza F, Guntinas-Lichius O, Petersen I (2010) HPV status in head and neck tumors. Pathol Res Pract 206: 229–234. 10.1016/j.prp.2009.11.007 20138710

[14] Pachmann K, Camara O, Kavallaris A, Krauspe S, Malarski N, Gajda M, et al (2008) Monitoring the response of circulating epithelial tumor cells to adjuvant chemotherapy in breast cancer allows detection of patients at risk of early relapse. J Clin Oncol 26: 1208–1215. 10.1200/JCO.2007.13.6523 18323545

[15] Pachmann UA, Hekimian K, Carl S, Ruediger N, Rabenstein C, Pachmann K (2011) Comparing Sequential Steps For Detection Of Circulating Tumor Cells: More Specific Or Just Less Sensitive?. WebmedCentral CANCER 2: WMC001490.

[16] Pachmann K, Camara O, Kavallaris A, Schneider U, Schünemann S, Höffken K. Quantification of the response of circulating epithelial cells (CEC) to neodadjuvant treatment of breast cancer: A new tool for therapy monitoring. Breast Cancer Research 7:R975–R979. 16280045 10.1186/bcr1328 PMC1410761

[17] Mockelmann N, Laban S, Pantel K, Knecht R (2014) Circulating tumor cells in head and neck cancer: clinical impact in diagnosis and follow-up. Eur Arch Otorhinolaryngol 271: 15–21. 10.1007/s00405-013-2391-6 23408023

[18] Hristozova T, Konschak R, Stromberger C, Fusi A, Liu Z, Weichert W et al (2011) The presence of circulating tumor cells (CTCss) correlates with lymph node metastasis in nonresectable squamous cell carcinoma of the head and neck region (SCCHN). Ann Oncol 22: 1878–1885. 10.1093/annonc/mdr130 21525401

[19] Weller P, Nel I, Hassenkamp P, Gauler T, Schlueter A, Lang S et al (2014) Detection of circulating tumor cell subpopulations in patients with head and neck squamous cell carcinoma (HNSCC). PLoS One 9: e113706 10.1371/journal.pone.0113706 25479539 PMC4257624

[20] Wikner J, Grobe A, Pantel K, Riethdorf S (2014) Squamous cell carcinoma of the oral cavity and circulating tumour cells. World J Clin Oncol 5: 114–124. 10.5306/wjco.v5.i2.114 24829858 PMC4014783

[21] Gröbe A, Blessmann M, Hanken H, Friedrich RE, Schön G, Wikner J, et al (2014) Prognostic relevance of circulating tumor cells in blood and disseminated tumor cells in bone marrow of patients with squamous cell carcinoma of the oral cavity. Clin Cancer Res 20: 425–433. 10.1158/1078-0432.CCR-13-1101 24218516

[22] Buglione M, Grisanti S, Almici C, Mangoni M, Polli C, Consoli F, et al (2012) Circulating tumour cells in locally advanced head and neck cancer: preliminary report about their possible role in predicting response to non-surgical treatment and survival. Eur J Cancer 48: 3019–3026. 10.1016/j.ejca.2012.05.007 22682019

[23] Nichols AC, Lowes LE, Szeto CC, Basmaji J, Dhaliwal S, Chapeskie C, et al (2012) Detection of circulating tumor cells in advanced head and neck cancer using the CellSearch system. Head Neck 34: 1440–1444. 10.1002/hed.21941 22076949

[24] Hsieh JC, Lin HC, Huang CY, Hsu HL, Wu TM, et al. (2014) Prognostic value of circulating tumor cells with podoplanin expression in patients with locally advanced or metastatic head and neck squamous cell carcinoma. Head Neck, 10.1002/hed.23779 [Epub ahead of print]. 24844673

[25] Mirghani H, Amen F, Moreau F, Lacau St Guily J (2015) Do high-risk human papillomaviruses cause oral cavity squamous cell carcinoma? Oral Oncol; 51: 229–236. 10.1016/j.oraloncology.2014.11.011 25488179

[26] Hitt R, López-Pousa A, Martínez-Trufero J, Escrig V, Carles J, Rizo A, et al (2005) Phase III study comparing cisplatin plus fluorouracil to paclitaxel, cisplatin, and fluorouracil induction chemotherapy followed by chemoradiotherapy in locally advanced head and neck cancer. J Clin Oncol 23: 8636–8645. 16275937 10.1200/JCO.2004.00.1990

[27] Lorch JH, Goloubeva O, Haddad RI, Cullen K, Sarlis N, Tishler R, et al (2011) Induction chemotherapy with cisplatin and fluorouracil alone or in combination with docetaxel in locally advanced squamous-cell cancer of the head and neck: long-term results of the TAX 324 randomised phase 3 trial. Lancet Oncol 12: 153–159. 10.1016/S1470-2045(10)70279-5 21233014 PMC4356902

[28] Jatana KR, Balasubramanian P, Lang JC, Yang L, Jatana CA, White E, et al (2010) Significance of circulating tumor cells in patients with squamous cell carcinoma of the head and neck: initial results. Arch Otolaryngol Head Neck Surg 136: 1274–1279. 10.1001/archoto.2010.223 21173379 PMC3740520

[29] Tinhofer I, Konschak R, Stromberger C, Raguse JD, Dreyer JH, Jöhrens K, et al (2014) Detection of circulating tumor cells for prediction of recurrence after adjuvant chemoradiation in locally advanced squamous cell carcinoma of the head and neck. Ann Oncol 25: 2042–2047. 10.1093/annonc/mdu271 25057171

[30] Kusukawa J1, Suefuji Y, Ryu F, Noguchi R, Iwamoto O, Kameyama T (2000) Dissemination of cancer cells into circulation occurs by incisional biopsy of oral squamous cell carcinoma. J Oral Pathol Med 29: 303–307. 10947245 10.1034/j.1600-0714.2000.290703.x

[31] Liu Z, Jiang M, Zhao J, Ju H (2007) Circulating tumor cells in perioperative esophageal cancer patients: quantitative assay system and potential clinical utility. Clin Cancer Res 13: 2992–2997. 17505001 10.1158/1078-0432.CCR-06-2072

[32] Hashimoto M, Tanaka F, Yoneda K, Takuwa T, Matsumoto S, Okumura Y, et al (2014) Significant increase in circulating tumour cells in pulmonary venous blood during surgical manipulation in patients with primary lung cancer. Interact Cardiovasc Thorac Surg 18: 775–783. 10.1093/icvts/ivu048 24618055

[33] Nesteruk D, Rutkowski A, Fabisiewicz S, Pawlak J, Siedlecki JA, Fabisiewicz A. (2014) Evaluation of prognostic significance of circulating tumor cells detection in rectal cancer patients treated with preoperative radiotherapy: prospectively collected material data. Biomed Res Int: 712827. 10.1155/2014/712827 PMC4070579 25006584

[34] Perrone F, Bossi P, Cortelazzi B, Locati L, Quattrone P, Pierotti MA, et al (2010) TP53 mutations and pathologic complete response to neoadjuvant cisplatin and fluorouracil chemotherapy in resected oral cavity squamous cell carcinoma. J Clin Oncol 28: 761–766. 10.1200/JCO.2009.22.4170 20048189

[35] Feng Z, Guo W, Zhang C, Xu Q, Zhang P, Sun J, et al (2011) CCND1 as a predictive biomarker of neoadjuvant chemotherapy in patients with locally advanced head and neck squamous cell carcinoma. PLoS One 6(10): e26399 10.1371/journal.pone.0026399 22065993 PMC3204964

[36] Zhu DW, Liu Y, Yang X, Yang CZ, Ma J, Yang X, et al (2013) Low Annexin A1 expression predicts benefit from induction chemotherapy in oral cancer patients with moderate or poor pathologic differentiation grade. BMC Cancer 13:301 10.1186/1471-2407-13-301 23786757 PMC3702430

