# Supplementary material for: Prognostic Role of Circulating Tumor Cells during Induction Chemotherapy Followed by Curative Surgery Combined with Postoperative Radiotherapy in Patients with Locally Advanced Oral and Oropharyngeal Squamous Cell Cancer
Source: PLoS One. 2015 Jul 17;10(7):e0132901. doi: 10.1371/journal.pone.0132901 (PMC4505900; doi:10.1371/journal.pone.0132901)
Supplement: S1 Table — (DOCX) [file pone.0132901.s001.docx]

**S1 Table**

| Association between CTC at baseline and maximal CTC during therapy, respectively, to other clinicopathologic characteristics. | | |
| --- | --- | --- |
|  | CTC at baseline (<>median) | Maximal CTC during therapy  (<>median) |
| Parameter | p | p |
| Gender | 1.000 | 0.212 |
| Age (<>median of 58 years) | 1.000 | 0.058 |
| Localization, oral cavity vs oropharynx | 0.225 | **0.022** |
| cT Stage (T2 vs T3/T4) | 0.686 | 0.744 |
| cN Stage (N0 vs N+) | 0.541 | 0.548 |
| HPV status (HPV positive vs negative) | 0.303 | 0.062 |
| Pathological response (CR vs not CR) | 1.000 | 1.000 |
| Therapy arm (Arm A vs B) | 0.699 | 0.744 |
| CTC at baseline (<>median) | **---** | **0.023** |
| CTC maximal during therapy (<>median) | **0.023** | **---** |
| CTC increase after IC (yes vs no) | 0.387 | 0.714 |
| CTC increase after surgery (yes vs no) | 0.346 | 0.512 |
| CTC increase after radiotherapy (yes vs no) | 0.212 | 0.657 |
| Recurrence (yes vs no) | **0.014** | 0.429 |
| Death | 1.000 | **0.028** |
